# Circulating inflammatory and neurotrophic markers as moderators and/or mediators of cognitive remediation outcome in people with bipolar disorders

**DOI:** 10.1192/bjo.2024.818

**Published:** 2024-12-05

**Authors:** Rebecca Strawbridge, Dimosthenis Tsapekos, Allan H. Young

**Affiliations:** Department of Psychological Medicine, Institute of Psychiatry, Psychology & Neuroscience, King's College London, UK

**Keywords:** Bipolar, cognition, cognitive remediation, inflammation, biomarker

## Abstract

**Background:**

Immune dysregulation appears involved in affective disorder pathophysiology. Inflammatory biomarkers have been linked with the cognitive impairment observed in people with bipolar disorders and as such are candidate markers that may improve with, and/or predict outcomes to, cognitive remediation therapies (CRT).

**Aims:**

Nine candidate biomarkers were examined as putative mediators and/or moderators to improvements following CRT compared with treatment as usual (TAU) from a randomised controlled trial.

**Method:**

Euthymic adults with bipolar disorders who had been randomised to CRT (*n* = 23) or TAU (*n* = 21) underwent blood testing before and after a 12 week intervention period. Five cytokines and four growth factor proteins, selected *a priori*, were examined in association with global cognition and psychosocial functioning outcomes.

**Results:**

CRT attenuated a reduction in the brain-derived neurotrophic factor (BDNF), basic fibroblast growth factor and vascular endothelial growth factor-C compared to TAU. For the BDNF, lower baseline levels predicted better functional outcomes across the sample but was more pronounced in TAU versus CRT participants and indicated larger CRT effects in those with a higher BDNF. A moderation effect was also apparent for tumour necrosis factor-β and interleukin-16, with greater CRT versus TAU effects on functioning for participants with lower baseline levels.

**Conclusions:**

Although preliminary, results suggest that CRT may exert some protective biological effects, and that people with lower levels of neurotrophins or cytokines may benefit more from CRT. We note an absence of associations with cognitive (versus functional) outcomes. These findings require further examination in large well-controlled studies.

Cognitive impairment contributes substantially to the extensive disability burden of bipolar disorders, such that it is now being prioritised as a key target of treatment for cognitive and functional recovery for patients.^1^ Cognitive remediation therapies represent, to date, the most rigorously investigated intervention to enhance cognition and function in this population.^2^ The ‘Cognitive Remediation in Bipolar’ (CRiB) study^3^ (to our knowledge, the largest study to date; *n* = 80, although considered a feasibility trial)^4^ found that participants undergoing cognitive remediation demonstrated improvements compared to those randomised to usual care in blinded assessed measures of IQ, working memory, executive function, global cognition, verbal recall, processing speed and psychosocial functioning, many both immediately following the 12 week intervention period and a further 12 weeks afterwards.^4,5^ Secondary examinations of this pilot study have provided preliminary evidence that a range of non-biological participant characteristics did not moderate cognitive remediation effects on global cognitive or functional outcomes,^6^ but that cognitive improvements after cognitive remediation partially mediated subsequent improvements to psychosocial functioning, albeit with a large proportion of this effect remaining unexplained.^7^ Consequently, it has been proposed that the identification of markers that both predict cognitive (and functional) outcomes in people undergoing cognitive remediation, and/or that could represent therapeutic ingredients (or moderators) of improvement during therapy, may have substantial value in the future implementation and personalisation of interventions.^8^ Candidate markers of cognitive improvement may be psychological (e.g. metacognitive awareness) or biological.^8^ Particularly promising biological domains include neurotrophic and immune systems: both have been extensively associated with cognitive functioning both outside of, and within, populations with bipolar disorder,^9,10^ and acute pro-inflammatory challenges (e.g. lipopolysaccharide) are known to induce impairments to cognition.^11^ A previous examination of the CRiB study identified previously established novel proteins that were associated with cognitive performance/impairment at baseline in this study.^12^ The established markers here comprise some neurotrophic factors known to play a role in hippocampal neurogenesis and relatedly memory synthesis (brain-derived neurotrophic factor (BDNF), basic fibroblast growth factor (bFGF)) and other growth factors whose upregulation of growth and maintenance of cells appear protective against cognitive impairment (vascular endothelial growth factor-C (VEGF-C) and placental growth factor (PlGF), as well as the regulatory cytokine interleukin-7 (IL-7)). Less established are interleukin-6 (IL-6), a pro-inflammatory cytokine that conversely has been associated with poorer outcomes generally, particularly in people with affective disorders, tumour necrosis factor-β (TNF-β), interleukin-16 (IL-16) and macrophage inflammatory protein-1β (Mip1β), which also act as generally pro-inflammatory markers, albeit with inconsistent findings in affective disorder psychopathology.^12^ To our knowledge, no studies have yet explored these as putative candidates of cognitive outcome following intervention (compared with a no-intervention control).

## Aims and objectives

The present study was a preliminary exploration of these nine candidate proteins in association with cognitive outcomes following intervention in the trial. These were as above: IL-6, IL-16, IL-7, TNF-β, Mip1β, VEGF-C, PlGF, bFGF and BDNF. Specifically, we sought to analyse the following:

- whether biomarker levels at baseline (week 0) predicted cognitive outcomes (week 13) across the full sample of participants, regardless of intervention;
- whether biomarker levels at baseline (week 0) predicted cognitive outcomes to cognitive remediation relative to usual care (week 13), that is, moderators of outcome;
- whether biomarker levels changed between baseline (week 0) and post-intervention (week 13), and whether this was significantly different between intervention groups;
- whether changes in biomarker levels from baseline (week 0) to post-intervention (week 13) predicted longer-term cognitive outcomes (week 25) to cognitive remediation, that is, mediators of outcome.

As a secondary exploratory outcome, a recovery outcome of psychosocial functioning (Functioning Assessment Short Test (FAST)) was used for each of the above comparisons.

As a preliminary study, it was not considered appropriate to pre-specify hypotheses. Since most of these proteins were considered candidates based on being negatively associated with cognitive impairment (i.e. lower levels alongside greater impairment), it may have been anticipated that those with lower levels might have had better outcomes with cognitive remediation versus usual care, that cognitive remediation may have increased their levels and that these increases could have been associated with subsequent cognitive gains. The opposite direction of effect could have been anticipated with IL-6, based on previous findings.^12^

## Method

### Design

This study is a secondary analysis from the randomised controlled CRiB study. Previous reports provide high-level detail of the study's methodology and findings.^3,5^ The present examination is from a subsample of 44 individuals who underwent optional blood draws for biomarker analysis at baseline (week 0) and following intervention with either cognitive remediation or usual care (week 13).^12^

### Participants

Participants were required to have a diagnosis of bipolar disorder (type I or II), have been in a euthymic affective state for at least 1 month, be aged 18–65 years, fluent in English and without current substance use, personality or impairing organic neurological disorders. Diagnoses were validated using the MINI-International Neuropsychiatric Interview (MINI).^13^ Euthymia was established using the Newcastle Euthymia Protocol (scores ≤7 on the Hamilton Rating Scale for Depression (HRSD)^14^ and Young Mania Rating Scale (YMRS)^15^ at two timepoints that covered 1 month before inclusion).

### Procedure

All procedures contributing to this work comply with the ethical standards of the relevant national and institutional committees on human experimentation and with the Helsinki Declaration of 1975, as revised in 2008. All procedures involving human participants/patients were approved by the UK's Health Research Authority and London City Road & Hampstead National Health Service (NHS) Research Ethics Committee (id. 15/LO/1557). Before recruitment, approvals were obtained (as per above) and the trial registered with ISRCTN (id. 32290525). The first participant was recruited on 19 February 2016. Participants were recruited through healthcare services and community advertisement. All participants provided written fully informed consent before taking part. Blood samples were collected within the same session as primary trial baseline (week 0) and post-intervention (week13) assessments; no blood tests were conducted at the final follow-up (week 25) assessment, although cognitive and functional outcomes were measured at the latter timepoint.

### Measures

The cognitive measure assessed in the current study was a continuous composite measure of ‘global cognition’ from eight measures included in the battery, as described previously.^12^ These included tests of processing speed, working memory, episodic learning and memory and executive functioning; four of these are used individually as key individual cognitive domains (see section ‘Statistical analyses’), that is, a digit symbol substitution test for processing speed,^16^ digit span for working memory,^16^ verbal paired associates II for episodic memory^17^ and the hotel test of executive function.^18^

Biological measures were derived from extracting plasma after 5 mL blood was taken and centrifugation. Samples were stored at −80°C degrees until they were thawed for assay. A Meso Scale Discovery (MSD) V-Plex kit (Meso Scale Diagnostics, Maryland, USA) was used in duplicate, following manufacturer processes, to determine all biomarker levels, with high detection sensitivity. The biomarkers examined in the current study comprise IL-6, IL-7, IL-16, TNF-β, Mip1β, VEGF-C, PlGF, bFGF and BDNF. Levels are expressed throughout as picograms per millilitre (pg/ml).

The only other non-biological measures used in this study were (1) psychosocial functioning (FAST)^19^ as a secondary outcome and three post-hoc potential covariates, that is, (2) the number of psychotropic medications taken, (3) health-related quality of life (EuroQol (EQ5D) score^20^) and (4) current smoking (yes/no).

### Statistical analyses

Protein levels were transformed (log base 10). Bootstrapping of 1000 samples was employed on all regression models. Across analyses, model assumptions for collinearity were checked (Durbin–Watson) with a *P*-value <0.05 considered significant. Analyses were conducted in SPSS version 28 (IBM) for Windows with moderation and mediation analyses employing the PROCESS macro and procedures.^21^ In light of the small sample size and exploratory nature of this study, no covariates (other than baseline measures of outcome) were included in the analyses described below (primary analyses), although these were subsequently re-run including factors that had previously been associated with biomarkers in this study, namely smoking, health-related quality of life and number of medications taken.

Objective 1: Multivariable linear regressions were undertaken for the whole sample, each with global cognition composite at week 13 and separately at week 25 as dependent variables; independent variables included were the baseline biological marker (‘predictor’) in addition to baseline global cognition composite as covariate.

Objective 2: Moderation analyses were undertaken to examine whether protein levels differentially predicted outcomes (separately for cognition and functioning at weeks 13 and 25) between people randomised to cognitive remediation versus treatment as usual (TAU). Models included the baseline biological marker (‘moderator’) and group allocation (‘independent variable’), with their interaction indicating differential prediction of outcome (cognitive/functional outcome as the dependent variable and baseline measure of outcome as the covariate).

Objective 3: 2 × 2 mixed-factorial analyses of variance (ANOVAs) were conducted to assess protein changes occurring between baseline (week 0) and post-intervention (week 13) (within-subjects), and between participants randomised to cognitive remediation versus TAU (time by group interaction).

Objective 4: Finally, we examined the association between changes in inflammatory proteins and cognitive/functional outcomes. First, correlations between change scores in these variables were checked at post-intervention (week 13). Then, mediation analyses were conducted to assess whether inflammatory proteins at post-intervention (week 13; adjusted for baseline levels) mediated the relationship between treatment (cognitive remediation versus TAU) and cognitive/functional outcomes subsequently at week 25 (adjusted for baseline measures of outcome). These analyses were conducted only for markers where there was a significant treatment effect emerging from objective 3, and additionally for the BDNF in examining outcomes to key individual cognitive tests (of processing speed, working memory, episodic memory and executive functioning, as previously^6^). This is based on extensive attention on the BDNF (in contrast to other proteins) as an established marker of cognition in schizophrenia, with evidence from multiple studies suggesting BDNF increases in response to therapy are associated with cognitive gains following successful pharmacological^22,23^ and cognitive training^24^ intervention, and subsequent reviews supporting this idea.^25,26^

## Results

Sample characteristics remain as previously described:^12^ 70.5% of participants were female, 59% had bipolar disorder type I, and the sample had an average age of 43.7 (s.d. = 12.8) and average body mass index (BMI) of 27.8 (s.d. = 6.1). The mean standardised global cognition score at baseline was −0.186 (s.d. = 0.609), at week 13 it was 0.082 (s.d. = 0.659) and at week 25 it was 0.303 (s.d. = 0.704). Table 1 contains data on biological and non-biological measures at each timepoint, separated by intervention group. No biological or non-biological variables had any missing values. Compared with the full study sample, this subsample had comparable (*P* < 0.05) difference in age, gender, BMI, education years and premorbid IQ, subsyndromal symptom severity at each timepoint, subjective cognitive complaints, cognitive performance and functional capacity (FAST) scores at each timepoint. Detailed characteristics of the full sample before and after cognitive remediation/TAU, including changes in cognition and functioning, have been presented elsewhere.^4–8^

**Table 1:** All study measures by timepoint and participant group

Variable		All (*N* = 44)	Cognitive remediation (*n* = 23)	TAU (*n* = 21)
*Binary variables*		*n* (%)	*n* (%)	*n* (%)
Gender	Female	31 (70)	17 (74)	14 (67)
	Male	13 (30)	6 (26)	7 (33)
Bipolar subtype	Type I	26 (63)	13 (57)	13 (62)
	Type II	18 (37)	10 (43)	8 (38)
Smoke	Yes	12 (27)	7 (30)	5 (24)
	No	32 (73)	16 (70)	16 (76)
*Continuous variables*		*Mean (s.d.)*	*Mean (s.d.)*	*Mean (s.d.)*
Age		43.68 (12.84)	43.57 (13.16)	43.81 (12.81)
Global cognition standardised score (higher score = higher performance)	Week 0	−0.19 (0.61)	−0.21 (0.58)	−0.164 (0.65)
	Week 13	0.08 (0.66)	0.228 (0.60)	−0.077 (0.70)
	Week 25	0.30 (0.70)	0.524 (0.64)	0.061 (0.71)
Functioning score (higher score = *lower* functioning)	Week 0	20.80 (9.62)	22.83 (10.81)	18.57 (7.79)
	Week 13	18.87 (9.41)	13.60 (10.48)	19.67 (8.27)
	Week 25	18.82 (9.37)	17.48 (9.63)	20.29 (9.07)
_log_BDNF	Week 0	3.21 (0.27)	3.15 (0.24)	3.27 (0.29)
	Week 13	3.14 (0.26)	3.17 (0.29)	3.10 (0.22)
_log_bFGF	Week 0	1.25 (0.43)	1.19 (0.44)	1.33 (0.41)
	Week 13	1.18 (0.38)	1.20 (0.43)	1.15 (0.31)
_log_IL-16	Week 0	2.13 (0.14)	2.14 (0.13)	2.11 (0.14)
	Week 13	2.10 (0.12)	2.12 (0.13)	2.07 (0.11)
_log_IL-6	Week 0	−0.19 (0.37)	−0.14 (0.44)	−0.25 (0.28)
	Week 13	−0.19 (0.38)	−0.14 (0.45)	−0.24 (0.29)
_log_IL-7	Week 0	0.40 (0.31)	0.34 (0.30)	0.47 (0.31)
	Week 13	0.34 (0.28)	0.34 (0.31)	0.33 (0.25)
_log_Mip1β	Week 0	1.69 (0.17)	1.66 (0.16)	1.72 (0.19)
	Week 13	1.67 (0.16)	1.66 (0.17)	1.68 (0.15)
_log_PlGF	Week 0	0.73 (0.12)	0.74 (0.11)	0.71 (0.14)
	Week 13	0.72 (0.13)	0.73 (0.13)	0.70 (0.13)
_log_TNF-β	Week 0	−0.60 (0.38)	−0.62 (0.39)	−0.58 (0.39)
	Week 13	−0.65 (0.35)	−0.68 (0.38)	−0.61 (0.32)
_log_VEGF-C	Week 0	1.78 (0.22)	1.76 (0.20)	1.81 (0.24)
	Week 13	1.68 (0.28)	1.73 (0.25)	1.63 (0.32)

TAU, treatment as usual; BDNF, brain-derived neurotrophic factor; bFGF, basic fibroblast growth factor; IL-16, interleukin-16; IL-6, interleukin-6; IL-7, interleukin-7; Mip1β, macrophage inflammatory protein-1β; PlGF, placental growth factor; TNF-β, tumour necrosis factor-β; VEGF-C, vascular endothelial growth factor-C.

### Proteomic predictors of subsequent outcome

These biomarkers unanimously did not predict cognitive outcomes at both timepoints across the sample. As presented in Table 2, participants with lower baseline levels of BDNF and IL-7 had a better functional outcome (lower FAST scores, adjusted for baseline scores) at both weeks 13 and 25. The same pattern was identified for the bFGF and VEGF-C for week 13 (but not week 25) functional outcomes. The significance appeared stronger (at *P* < 0.01) for the BDNF (*P* = 0.005; *P* = 0.007), the bFGF (*P* = 0.003) and VEGF-C (*P* = 0.009); less so for IL-7 (*P* = 0.013; *P* = 0.036), whose association at week 25 became non-significant when adjusting for smoking, medication and quality of life. Conversely, a non-significant association in the unadjusted model for IL-6 prediction of week 25 functioning became significant when adjusting for these covariates (with the same direction of effect; *P* = 0.033).

**Table 2:** Proteomic predictors of outcome

	Week 13 cognition				Week 25 cognition				Week 13 function				Week 25 function			
	*b*^c^	95% CI		*P*	*b*^c^	95% CI		*P*	*b*^c^	95% CI		*P*	*b*^c^	95% CI		*P*
BDNF	−0.104	−0.607	0.189	0.598	−0.279	−0.886	0.180	0.278	**9.780**	**2.756**	**15.597**	**0.005**	**8.810**	**2.701**	**14.509**	**0.007**
bFGF	−0.015	−0.266	0.244	0.911	0.048	−0.272	0.451	0.804	**4.928**	**1.640**	**8.497**	**0.003**	3.038	−1.660	6.813	0.178
IL-6	0.103	−0.185	0.401	0.526	0.062	−0.359	0.440	0.770	0.810	−3.202	6.840	0.768	3.533	−1.654	9.369	0.182^a^
IL-7	−0.183	−0.546	0.150	0.300	−0.173	−0.673	0.368	0.493	**7.064**	**1.751**	**11.962**	**0.013**	**6.169**	**−0.320**	**11.471**	**0.036**^b^
IL-16	0.289	−0.548	1.007	0.462	0.158	−0.969	1.154	0.790	0.636	−13.184	10.649	0.911	−2.715	−19.444	10.030	0.725
Mip1β	0.246	−0.208	0.847	0.376	−0.471	−1.134	0.421	0.230	2.891	−10.285	15.159	0.644	−0.708	−15.065	10.049	0.901
PlGF	0.477	−0.330	1.392	0.272	0.210	−1.025	1.454	0.739	3.104	−8.480	15.506	0.593	−2.124	−17.975	12.449	0.769
TNF-β	0.064	−0.197	0.359	0.646	0.124	−0.243	0.619	0.569	1.073	−3.427	5.236	0.650	1.702	−3.512	6.809	0.526
VEGF-C	−0.133	−0.783	0.364	0.654	−0.156	−0.935	0.569	0.669	**9.177**	**2.400**	**15.953**	**0.009**	7.102	−1.618	14.360	0.077

BDNF, brain-derived neurotrophic factor; bFGF, basic fibroblast growth factor; IL-6, interleukin-6; IL-7, interleukin-7; IL-16, interleukin-16; Mip1β, macrophage inflammatory protein-1β; PlGF, placental growth factor; TNF-β, tumour necrosis factor-β; VEGF-C, vascular endothelial growth factor-C.

a. When adjusting for smoking, number of medications and health-related quality of life, IL-6 contributed significantly to week 25 functioning: *b* = 6.203 [95% 1.190–12.957], *sb* = 0.244, *P* = 0.033.

b. When adjusting for smoking, number of medications and health-related quality of life, this was no longer significant (*P* = 0.091).

c. Beta coefficient of predictor variable.

Higher cognitive scores indicate improved performance; lower functioning scores indicate improved function; thus, for cognition, a positive beta represents higher protein levels predicting a more pronounced improvement and, for functioning, a positive beta represents lower protein levels predicting a more pronounced improvement.

Bold text denotes effects significant at *P* > 0.05.

### Proteomic moderators of outcome to cognitive remediation versus TAU

**Fig. 1:**
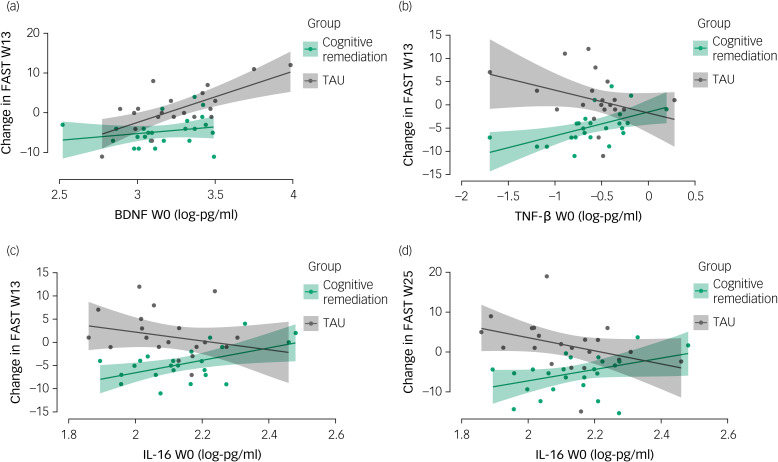
Moderation effect of functional outcome, with greater functional outcomes (reduction in Functioning Assessment Short Test (FAST)) for participants randomised to treatment as usual (TAU) relative to cognitive remediation with (a) lower baseline levels of brain-derived neurotrophic factor (BDNF) (week 13 outcome), (b) higher baseline levels of tumour necrosis factor-β (TNF-β) (week 13 outcome), (c) higher baseline levels of interleukin-16 (IL-16) at week 13 and (d) at week 25. Protein levels in log-pg/ml. W0, week 0; W13, week 13; W25, week 25.

As above, no biomarkers moderated cognitive outcomes in the trial. While a lower BDNF predicted better functional outcomes in the sample as a whole (see above), this effect was more pronounced in those randomised to TAU relative to cognitive remediation at week 13 (*P* = 0.016). The opposite direction of effect was observed for TNF-β at week 13 (*P* = 0.018) and for IL-16 at both weeks 13 (*P* = 0.021) and 25 (*P* = 0.008). See Fig. 1 and Table 3 for results of all moderation analyses.

**Table 3:** Proteomic moderators of outcome

	Week 13 cognition				Week 25 cognition				Week 13 function				Week 25 function			
	*b*^a^	95% CI		*P*	*b*^a^	95% CI		*P*	*b*^a^	95% CI		*P*	*b*^a^	95% CI		*P*
BDNF	−0.216	−0.986	0.555	0.574	0.081	−1.004	1.165	0.881	**−11.780**	**−21.281**	**−2.279**	**0.016**	−1.574	−14.711	11.563	0.810
bFGF	−0.280	−0.737	0.177	0.223	−0.096	−0.744	0.552	0.766	0.079	−6.217	6.374	0.980	1.517	−6.566	9.600	0.706
IL-6	−0.251	−0.853	0.351	0.404	0.156	−0.694	1.007	0.712	1.258	−7.606	10.122	0.776	4.797	−5.425	15.019	0.348
IL-7	−0.084	−0.739	0.571	0.796	0.295	−0.619	1.209	0.518	−1.641	−10.899	7.617	0.722	1.866	−9.773	13.506	0.747
IL-16	−0.112	−1.531	1.308	0.874	0.317	−1.675	2.309	0.749	**23.238**	**3.765**	**42.711**	**0.021**	**31.560**	**8.663**	**54.457**	**0.008**
Mip1β	0.031	−1.083	1.144	0.956	0.308	−1.284	1.900	0.698	−7.843	−24.661	8.975	0.351	−4.260	−24.364	15.843	0.671
PlGF	−0.115	−1.704	1.474	0.885	−0.504	−2.746	1.738	0.652	18.178	−4.536	40.893	0.114	21.003	−6.480	48.485	0.130
TNF-β	−0.016	−0.540	1.498	0.949	0.662	−0.023	1.348	0.579	**8.999**	**1.599**	**16.400**	**0.018**	6.982	−2.265	16.228	0.135
VEGF-C	−0.717	−1.580	0.146	0.101	0.028	−1.225	1.281	0.964	1.779	−10.445	14.002	0.770	6.887	−8.392	22.165	0.368

BDNF, brain-derived neurotrophic factor; bFGF, basic fibroblast growth factor; IL-6, interleukin-6; IL-7, interleukin-7; IL-16, interleukin-16; Mip1β, macrophage inflammatory protein-1β; PlGF, placental growth factor; TNF-β, tumour necrosis factor-β; VEGF-C, vascular endothelial growth factor-C.

a. Beta coefficient of predictor variable.

Higher cognitive scores indicate improved performance; lower functioning score indicate improved performance. Here cognitive remediation is coded as 1 and treatment as usual (TAU) as 0; thus, for cognition, a positive beta represents higher protein levels associated with a more pronounced improvement in the cognitive remediation versus TAU group and, for functioning, a positive beta represents lower protein levels associated with a more pronounced improvement in the cognitive remediation versus TAU group.

Bold text denotes effects significant at *P* > 0.05.

### Proteomic changes during cognitive remediation versus TAU

**Fig. 2:**
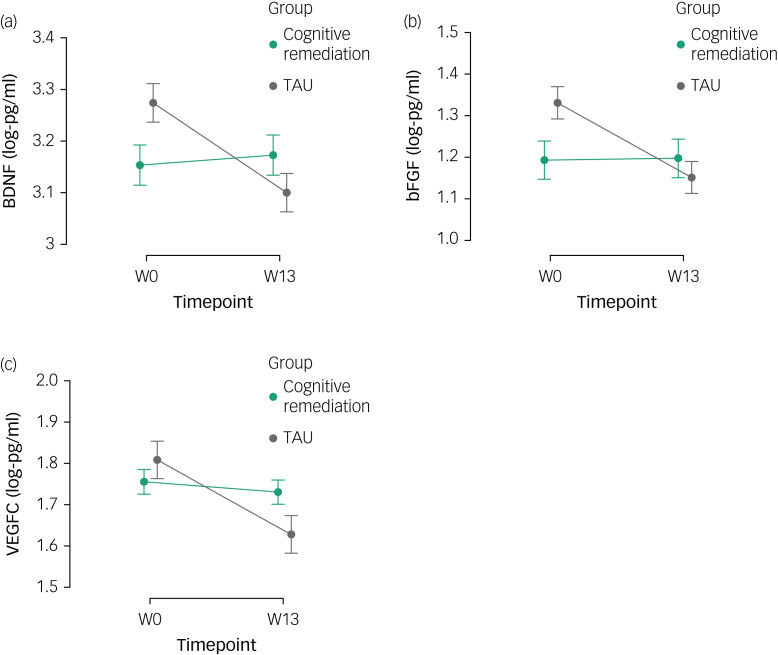
Protein changes during the intervention period with differences between participants randomised to cognitive remediation and treatment as usual (TAU), that is, (a) brain-derived neurotrophic factor (BDNF), (b) basic fibroblast growth factor (bFGF) and (c) vascular endothelial growth factor-C (VEGF-C). Protein levels in log-pg/ml. W0, week 0; W13, week 13.

Full results can be viewed in Table 4. IL-16 decreased significantly (*P* = 0.016) between the pre- and post-intervention timepoints but did not differ between groups. Three biomarkers showed both time (decreases) and time by group interactions, all marked by a decrease primarily in participants under TAU. As shown in Fig. 2, this was observed for the BDNF (where participants undergoing cognitive remediation showed a slight increase in levels; interaction *P* = 0.017; time *P* = 0.049), the bFGF (where cognitive remediation participants’ levels did not change overall; interaction *P* = 0.039; time *P* = 0.047) and VEGF-C (where cognitive remediation participants had a small decrease, in contrast with a marked decrease in TAU participants; interaction *P* = 0.046; time *P* = 0.010).

**Table 4:** Proteomic changes over time and between groups

	Time		Time × group	
	*F*^a^	*P*	*F*^a^	*P*
BDNF	**4**.**094**	**0**.**049**	**6**.**226**	**0**.**017**
bFGF	**4**.**183**	**0**.**047**	**4**.**564**	**0**.**039**
IL-6	0.022	0.884	0.001	0.981
IL-7	2.571	0.116	3.299	0.076
IL-16	**6**.**553**	**0**.**014**	1.970	0.168
Mip1β	1.224	0.275	0.641	0.428
PlGF	0.795	0.378	0.538	0.467
TNF-β	0.772	0.385	0.095	0.760
VEGF-C	**7**.**384**	**0**.**010**	**4**.**242**	**0**.**046**

BDNF, brain-derived neurotrophic factor; bFGF, basic fibroblast growth factor; IL-6, interleukin-6; IL-7, interleukin-7; IL-16, interleukin-16; Mip1β, macrophage inflammatory protein-1β; PlGF, placental growth factor; TNF-β, tumour necrosis factor-β; VEGF-C, vascular endothelial growth factor-C.

a. *F* statistic of ANOVA.

Degrees of freedom all 1, 42.

Bold text denotes effects significant at *P* > 0.05.

### Protein changes in mediating longer-term cognitive remediation outcomes

**Fig. 3:**
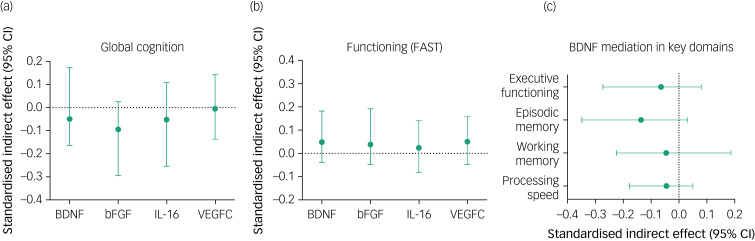
Indirect effects for different proteins as potential mediators of the treatment effect (cognitive remediation vs treatment as usual) on (a) global cognition and (b) functioning, and indirect effects for brain-derived neurotrophic factor (BDNF) as potential mediator of treatment effects on specific cognitive domains. bFGF, basic fibroblast growth factor; IL-16, interleukin-16; VEGF-C, vascular endothelial growth factor-C, FAST, Functioning Assessment Short Test.

Correlation analysis revealed significant associations between post-intervention improvement in functioning and increased levels of two proteins, the BDNF (*r* = −0.41, *P* = 0.006) and bFGF (*r* = −0.37, *P* = 0.013), across the sample. Mediation models were conducted to examine markers showing a significant time effect (above), that is, the BDNF, the bFGF, IL-16 and VEGF-C (week 13), as potential mediators for global cognitive and functional outcomes (week 25). No protein changes significantly mediated either subsequent cognitive or functional outcomes to cognitive remediation versus TAU. Indirect effects for these models are presented in Fig. 3. For the planned in-depth examination of the BDNF, again no full mediation was evident, despite cognitive remediation leading to increases in the BDNF for executive functioning. Details for correlation and mediation analyses are reported in Supplementary Tables 1 and 2 available at https://doi.org/10.1192/bjo.2024.818.

## Discussion

This study identified relevant proteomic markers to be largely associated with changes in functional but unexpectedly not cognitive outcomes following cognitive remediation therapy in people with euthymic bipolar disorders. Although an increase in the BDNF during the intervention period was correlated with increased functional benefits in the trial, no protein changes significantly mediated subsequent outcomes to cognitive remediation compared with TAU. Across comparisons, three markers – IL-6, Mip1β and PlGF – demonstrated no significant effects.

Interestingly, lower levels of growth factors (BDNF, bFGF, VEGF-C) and IL-7 as a regulatory cytokine preceded better functional outcomes. Moderation analyses demonstrated that for the BDNF, this association appeared mainly confined to the TAU group. For two other pro-inflammatory cytokines – IL-16 and TNF-β – a moderation effect in the reverse direction was identified, suggesting a magnification of cognitive remediation effects on functioning in people with lower baseline cytokine levels. IL-16 levels reduced during the intervention period similarly between groups. Three other markers showed reductions over time that were significantly more pronounced in TAU versus cognitive remediation participants; indeed, cognitive remediation appeared to mostly attenuate the reductions in protective growth factors – the BDNF, the bFGF and VEGF-C.

Overall, we interpret these findings as broadly indicative of a protective effect of cognitive remediation on declines in some proteins that are broadly associated with poorer cognitive functioning.^12^ This appears most pertinent for three neurotrophic growth factors – the BDNF, the bFGF and VEGF-C – for which similar results emerged: cognitive remediation attenuated a reduction in each of these compared to TAU, and lower baseline levels across the sample predicted better functional outcomes, although only for the BDNF did this appear specific to participants not undergoing cognitive remediation therapy. Only for the BDNF also was a reduction over time associated with poorer functional outcomes in the trial. The BDNF was indeed considered *a priori* the primary candidate marker examined, with the most substantial prior support suggesting a key role in illness-related cognitive impairment and improvement following therapy, mostly in individuals with psychoses, but also in those with bipolar disorders.^27^ In particular, previous studies have reported increases in the BDNF during clinical trials to be associated with superior cognitive gains after both pharmacological^22,23^ and non-pharmacological^24^ therapies.^25,26^ The latter aligns to some extent with our findings, although its profile in the current study is less of a rising BDNF in cognitively treated individuals and more of BDNF reductions in the absence of treatment or benefit. Relevant here is the lack of evidence thus far that cognitive remediation effectiveness is associated with age.^6^ Moreover, we observed no full mediation and no association with cognition itself, although in this trial cognitive and functional outcomes were closely related. The reason for BDNF reductions in this sample receiving TAU over a 3 month period is unclear; for example, even over several years a lack of significant change in serum BDNF has been reported.^28,29^ Conversely, a report of reduced BDNF manifesting over a long period in people who had a depressive illness compared with those without concluded that the BDNF can be considered a state marker of affective illness.^30^ However, our population were not experiencing depressive symptoms during the trial and subsyndromal depression severity did not differ between groups or timepoints.^5^

It would be also possible to consider a treatment effect modification effect by an unmeasured factor or factors. For instance, we could speculate that after the intervention period, participants in the TAU group experienced more subjective stress during the subsequent cognitive assessments and, although this may not have strongly affected their cognitive performance, it may have affected BDNF levels at the post-intervention timepoint. Although some previous studies have reported acute stress to reduce the BDNF,^31^ findings have also been reported in the opposite direction.^32^ Presence versus absence of the BDNF Val66Met polymorphism (rs6265) can clearly modify changes in the BDNF protein,^31^ and we were not able to measure this, although we note our similar findings between the BDNF and other similar neurotrophins. Nevertheless, the other markers examined can also be influenced by stress and all of these proteins are well-known to fluctuate according to numerous factors.^12^ Even for variables that were measured, we acknowledge that in the current study, potentially confounding factors were not substantively assessed. This was partly attributed to limited associations in our previous study (e.g. no association with BMI or subsyndromal symptoms). However, it was also partly because of the small sample size and exploratory nature of the study, and these will need further consideration in the future.

Increased stress in response to assessments at follow-up is one potential speculative reason outlined above why we may have observed greater proteomic changes overall in participants allocated to the TAU group. It may be of note that functional impairment (FAST) scores reduced by an average of approximately 5 points in the cognitive remediation group between weeks 0 and 25, while there was an average 2 point increase in the TAU group: whether a factor associated with allocation to TAU could explain both a (small) decline in functioning and a decline in neurotrophins will require a more substantial investigation. An unmeasured factor here may also explain the predictive effect of a higher BDNF before poorer functional outcome in TAU-randomised participants, which is as yet unexplained.

In total, a panel of 32 proteins were assayed from the CRiB study, but only nine were examined in this small exploratory study. These nine were selected because of their previous associations with cognitive functioning in a baseline analysis.^12^ Mip1β and IL-6 only showed a tentative relationship with cognition in the previous examination, and did not emerge as significant in the present study. Conversely, IL-7 and VEGF-C were strongly and consistently positively associated in the previous analysis and demonstrated similar findings to the BDNF in the current examination. Another neurotrophin – the PlGF – was also consistently associated with impairment previously but did not appear to be associated with cognitive remediation outcomes in this study.

The absence of significant associations with cognitive outcomes was somewhat unexpected. Both of the outcomes that we assessed comprise multiple domains, but this is particularly relevant for cognition. Our cognitive outcome measure was a composite score of several cognitive tests,^5^ which may have masked domain-specific associations. Many studies have compared biomarkers with specific cognitive domains and also reported associations that appear inconsistent between studies.^33,34^ It may be that comparisons with individual cognitive tests measured in this study would have yielded clearer findings, although this was not the case in our BDNF domain-specific mediation model that we tested. The frequent association between biomarkers and functional outcomes is somewhat surprising, given that these markers were considered candidate markers of outcomes to a therapy that principally focuses time on cognitive training. It is perhaps notable, though, that the functional outcome assessed (FAST) contains as one of its six domains ‘cognitive functioning’ (in addition to autonomy, occupational, financial, leisure and interpersonal relationships).^19^

### Further methodological considerations

As indicated above, and previously,^12^ this investigation was limited in statistical power because of the small sample size. To inform the design of future studies examining the mediating effect of proteomic biomarkers on cognitive and functional outcomes, we estimated sample sizes required to reach significance with 80% power based on indirect effect sizes detected in our study.^35^ The biomarker demonstrating the strongest indirect effect was examined for each outcome. For cognition, we estimated that a sample of 86 would be required to detect a mediating effect of the bFGF. For functioning, at least 322 participants would be needed to detect a significant mediating effect of VEGF-C. For the BDNF, this number ranged between 67 and 328 when considering mediating effects on different cognitive outcomes.

Limitations also included lack of adjustment for multiple comparisons, limited adjustment for potential confounding factors (including changes between groups in diet, exercise, stress and metabolism, which were not examined before and after the intervention) and lack of additional control groups. Conversely, there were further biomarker and cognitive measurements that could have been conducted and may have been informative. We only examined continuous cognitive outcomes, whereas it has previously been suggested that modelling, for example, cognitive impairment as a binary construct, may facilitate greater identification of biological relations.^12^

We would specifically mention that our analyses did not account for therapy characteristics (e.g. number of hours engaged) or other key factors that might have influenced therapy outcomes, for example, psychosocial stress. It is also notable that the decision was made to maximise inclusiveness of eligibility criteria for this secondary analysis because of the small sample size (e.g. meaning that, *a priori*, participants with autoimmune illnesses or taking anti-inflammatory medications were not excluded). Removing these had largely not affected results in our previous examination. Conversely, our study had the benefit of homogeneity of participant affective state, assessing biomarker and cognition closely in time and exploring a range of evidence-driven variable selection in terms of cytokines, unlike several previous studies.^34,36^

### Future implications

We identified preliminary evidence that peripheral growth factor proteins – the BDNF, the bFGF and VEGF – may moderate the effect of cognitive remediation in those with bipolar disorders, and potentially represent a mechanism for the effect of treatment on functioning. To delineate the true potential of candidate biomarkers to have utility in cognitive intervention, large-scale analyses similar to our own will be needed, where putative effect modifiers can be robustly accounted for in clinically assessing relevant candidate markers of meaningful patient outcomes to a variety of cognitive interventions. As yet, it remains unestablished whether biomarkers could predict cognitive improvement or decline trajectories irrespective of, or specific to, interventions. In addition to previously established markers (chiefly, BDNF), our work highlights novel candidates that may constitute targets for targeted cognitive intervention, for example, IL-7.^37^ Several important questions remain: Do relevant neurotropic or inflammatory proteins predict changes in cognition or function following cognitive remediation or without intervention? Do relevant neurotropic or inflammatory proteins change during cognitive remediation? Are relevant neurotropic or inflammatory proteins therapeutic mechanisms of cognitive remediation? In addition, which proteins – and outcomes – are relevant?

## Supporting information

Strawbridge et al. supplementary material Strawbridge et al. supplementary material

## Data Availability

Data availability requests should be submitted to the corresponding author.
