# Supplementary material for: Circulating inflammatory and neurotrophic markers as moderators and/or mediators of cognitive remediation outcome in people with bipolar disorders
Source: BJPsych Open. 2024 Dec 5;10(6):e225. doi: 10.1192/bjo.2024.818 (PMC11698213; doi:10.1192/bjo.2024.818)
Supplement: Strawbridge et al. supplementary material [file S2056472424008184sup001.docx]

**Supplementary Table 1: Proteomic correlates of post-treatment outcomes (N=44).**

|  | ***Global cognitive outcome*** | | ***Functional outcome*** | |
| --- | --- | --- | --- | --- |
|  | *r* | *p* | *r* | *p* |
| BDNF | 0.09 | 0.54 | **-0.41** | **<0.01** |
| bFGF | 0.11 | 0.47 | **-0.37** | **0.01** |
| IL-16 | -0.07 | 0.65 | -0.04 | 0.77 |
| VEGF-C | 0.06 | 0.70 | -0.29 | 0.06 |

**Supplementary Table 2: Proteomic mediators of subsequent outcomes (N=44).**

|  | **Indirect pathway Tx group -> protein -> outcome** | | |  |
| --- | --- | --- | --- | --- |
|  | *beta* | *95% CI* | |  |
| ***Global cognitive outcome*** | | | |  |
| BDNF | -0.049 | -0.164 | 0.174 |  |
| bFGF | -0.093 | -0.294 | 0.026 |  |
| IL-16 | -0.052 | -0.254 | 0.109 |  |
| VEGF-C | -0.004 | -0.137 | 0.143 |  |
| ***Functional outcome*** | | | |  |
| BDNF | 0.046 | -0.044 | 0.181 |  |
| bFGF | 0.035 | -0.051 | 0.190 |  |
| IL-16 | 0.019 | -0.084 | 0.139 |  |
| VEGF-C | 0.048 | -0.050 | 0.156 |  |
| ***BDNF examinations of key cognitive outcomes*** | | | | |
| Processing speed (DSST) | | -0.044 | -0.177 | 0.051 |
| Working memory (digit span) | | -0.045 | -0.224 | 0.187 |
| Episodic memory (VPA) | | -0.135 | -0.348 | 0.032 |
| Executive function (hotel) | | -0.064 | -0.272 | 0.080 |
